# Attitudes of dermatological patients towards digital health interventions: a cross‐sectional survey and cluster analysis

**DOI:** 10.1111/ddg.70010

**Published:** 2026-04-16

**Authors:** Patrick Reinders, Matthias Augustin, Marina Otten

**Affiliations:** ^1^ Institute for Health Services Research in Dermatology and Nursing (IVDP) University Medical Center Hamburg‐Eppendorf (UKE) Hamburg Germany

**Keywords:** attitudes, dermatology, digital health, eHealth, patients, skin diseases

## Abstract

**Background:**

This study aimed to assess the acceptance of Germany's adoption of digital health interventions (DHI) among dermatology patientsand to identify patient clusters.

**Patients and Methods:**

A cross‐sectional, quantitative survey on acceptability and (future) usage of DHIs was conducted among patients with chronic dermatological conditions. Exploratory Factor Analysis (EFA) identified key constructs and patients were grouped using a two‐step clustering approach.

**Results:**

The EFA was conducted using data from 344 patients (mean age 52.5 years, SD 14.8; 74.0 % female) and identified four factors: “Acceptability of DHIs and Trust,” “Patients’ Digital Health Competencies,” “Digital Health Insecurities,” and “Positive Impact of DHIs.” Cluster analysis resulted in four groups: “Digital Sceptics” (n = 49, 15.6 %), “Cautious Adopters” (n = 106, 33.7 %), “Digital Enthusiasts” (n = 98, 31.1 %), “Adopters Unsure About Impact” (n = 62, 18.0 %). “Digital Enthusiasts” were significantly younger. Usage and acceptability varied among clusters, with “Digital Enthusiasts” and “Adopters Unsure About Impact” showing the highest rates. Overall, few patients used DHIs, including store‐and‐forward teledermatology (2.0 %–7.0 %) or electronic patient records (6.4 %–19.4 %).

**Conclusions:**

Patient clusters with varying attitudes towards DHIs were identified. Despite a general willingness to adopt DHIs, actual usage remains low. Systemic barriers such as availability and digital competencies need to be addressed.

## INTRODUCTION

Dermatology deals with a wide range of highly prevalent skin conditions, including common chronic skin diseases such as acne, psoriasis, atopic dermatitis, and skin cancer.^1^, ^2^ In Germany, approximately 27 % of adults are affected by at least one dermatological condition annually. This results in a high caseload, causing prolonged waiting times for appointments.^3^, ^4^ Moreover, the restricted capacity is considered to be intensified by the demographic transition.^4^, ^5^, ^6^, ^7^

Proposed as a solution for sustainable health care, digital health interventions (DHIs) aim to improve the quality of care, enhance efficiency, and empower patients.^8^, ^9^ In this study, DHIs refer to health services that use information and communication technologies for patients, consumers, or healthcare professionals.^9^, ^10^ In dermatology, many promising DHIs have already emerged,^11^ including artificial intelligence applications,^12^ teledermatology for referrals, diagnoses, or the monitoring of skin diseases,^13^, ^14^, ^15^ and patient‐oriented applications aimed at improving medication adherence.^16^ While the 2020 teledermatology consensus guideline supports the use of teledermatology for certain indications, it also emphasizes the need for more robust evidence to assess associated benefits and risks.^17^

Despite the presence of existing DHIs, healthcare digitalization in Germany lags behind that of other Western nations.^18^ In Dermatology, even in the height of the COVID‐19 pandemic only a limited number of dermatologists integrated DHIs into care.^19^, ^20^ In addition, the usage by patients with skin diseases is relatively low, for example only 10 % used an electronic patient record (ePA).^21^ The intricacies of the German healthcare system contribute to the complexity of low DHI implementation rates, involving factors such as the absence of interoperability, high standards of data protection, or the lack of incentives for clinics and outpatient practices.^22^

Other reasons for the low usage rate of DHIs described by dermatologists may be the low acceptance of DHI by patients and their limited digital competencies.^23^ Some publications, however, reveal a sufficient to high acceptance of DHI and sufficient digital competencies.^21^, ^24^, ^25^ However, studies are limited either by a small sample size, the focus on one dermatological indication, highly selected patients or a limited number of items to investigate patient acceptance in greater detail.

With our study, we seek to add to the existing literature by examining the acceptance and current/future usage of DHIs among patients with chronic skin diseases. Additional objectives include identifying distinct patient clusters based on their acceptance and exploring their associations with sociodemographic variables and DHI adoption.

## PATIENTS AND METHODS

The Consensus‐Based *Checklist for Reporting of Survey Studies* (CROSS) was used to report the study.^26^ The checklist is available in Online Supplement S1.

### Study design and questionnaire

In March 2022, an anonymous cross‐sectional survey was distributed among patients with chronic skin diseases in Germany. The survey was constructed based on results from previously conducted focus groups involving dermatologists, patients, and nurses as well as findings from existing literature. The objective of the focus group study was to explore the acceptability of, barriers to, and facilitators of DHIs in dermatology.^23^ Building upon these outcomes, we developed statements and matched them with questionnaires from the literature.^21^, ^27^, ^28^ Thereby, we developed 31 items related to acceptability (e.g., “I could imagine using more digital applications for my condition”). Based on the systematic literature mapping,^11^ eleven items on the potential future usage of specific DHIs were incorporated (e.g., teledermatology or electronic patient diary). Respondents rated all items on a five‐point Likert scale (1 – strongly disagree; 5 – strongly agree); the numbers were not visible while responding. In addition, the survey asked about the current usage of the same eleven specific DHIs, to be answered with “Yes”, “No”, or “I do not know”.

Sociodemographic items included age, sex, education, and postal code. The latter was used to allocate supplementary regional information (e.g., urban or rural classification) to the participants. The survey also included items about the patients’ dermatological conditions (such as psoriasis, atopic dermatitis, vitiligo, skin cancer, and hidradenitis suppurativa), duration since the onset of symptoms and diagnosis, self‐perceived health using the first item of the SF‐36,^29^ and details regarding the attending physician.

The questionnaire was pilot tested with members of the patient association *Deutscher Psoriasis Bund e.V*. (*engl*.: German psoriasis association) and adapted. The items of the final survey are provided in Online Supplement S2.

### Study population, recruitment, and data entry

To conduct an *exploratory factor analysis* (EFA), as described in the statistical analysis section, we aimed for a minimum sample size of 150 patients.^30^ To enhance the generalizability of our findings, we included patients diagnosed with several chronic skin diseases, including psoriasis, vitiligo, hidradenitis suppurativa, atopic dermatitis, and skin cancer. Patient recruitment was conducted online through various patient self‐help organizations and patient advocacy groups, namely the

- *Deutscher Psoriasis Bund* (German psoriasis association) for psoriasis (approximately 1,700 members invited via email),
- *Deutscher Vitiligo Bund* (German Vitiligo Association) for vitiligo (approximately 250 members invited via email),
- *MulleWupp e. V*. for hidradenitis suppurativa (post in Facebook‐group with approximately 900 members),
- *Deutscher Allergie und Asthmabund* (German Allergy and Asthma Association) for atopic dermatitis (approximately 1,000 members invited via email),
- *SHG Hautkrebs Hamburg.info* (Self‐help group skin cancer Hamburg.info) for skin cancer (approximately 400 members invited via email),
- and *Melanom‐Info Deutschland* (Melanoma‐Info Germany) for skin cancer (post in Facebook group with approximately 8,000 members).

The online survey was conducted via Unipark and terminated 12 weeks after the first organization invited its participants. To prevent multiple entries by a single patient, participation was monitored using Unipark's cookie system (no stored IP addresses).

### Ethics Approval

The study was submitted to the local psychological ethics committee (Lokale Psychologische Ethikkomission am Zentrum für Psychosoziale Medizin) and received approval (LPEK‐0407). It was conducted in adherence to the principles of Good Scientific Practice and the Declaration of Helsinki.^31^, ^32^

### Statistical Analysis

Following a descriptive analysis of all sociodemographic and geographic parameters, an exploratory factor analysis (EFA) (Method of rotation: Oblimin) was conducted on all 31 items related to acceptability for two primary reasons: *(1)* data reduction by identifying underlying factors and *(2)* using the factors to better describe the acceptance by patients.^33^ Adequacy for EFA was assumed with a Kaiser‐Meyer‐Olkin (KMO) criterion ≥ 0.8 and Bartlett's test of sphericity < 0.05. Factors with an eigenvalue ≥ 1.0 were considered and items were retained based on substantial factor loading (>|0.4|) on one factor, without strong cross‐loading onto other factors. Cross‐loading was defined as loadings (>|0.3|) on another factor, with a difference of less than 0.2 between the loading on the main factor and the strongest loading on any other factor.^34^ Internal consistencies of factors were assessed using Cronbach's alpha, with values > 0.7 considered acceptable, > 0.8 considered good, and > 0.9 considered excellent.

To detect distinct clusters among patients with chronic skin diseases varying in acceptability, a two‐step clustering approach combined of hierarchical and K‐means cluster analyses was employed. The mean scores (using Likert scale ratings: 1–5) of the factors served as input variables for both approaches. The hierarchical cluster method determined the optimal number of clusters, and subsequently, a K‐means cluster analysis was conducted based on this optimal number. To examine the differences among the identified clusters in terms of mean scores, a one‐way analysis of variance (ANOVA) was performed. To profile the identified clusters, each item from the EFA was binary coded (1: fully agree/agree; 0: partly, disagree, fully disagree). Then, a descriptive analysis was performed, examining each item for each cluster. Chi‐squared tests were applied for categorical variables to reveal associations between clusters and sociodemographic parameters. If more than 20 % of cells had a count below five, Fisher's exact tests (FET) were used. The one‐way ANOVA was employed for continuous variables. A significance level of 0.05 was used and missing rates were reported for all variables. No imputation or weighting methods were applied. SPSS Version 29 was used for all statistical analyses.

## RESULTS

A total of 344 patients with at least one of the pre‐defined dermatological conditions participated in the survey. They were on average 52.5 years old (standard deviation [SD] 14.8), with 74.0 % being female, 75.9 % residing in an urban county or city, and 58.9 % having a high level of education (Table 1). The five included dermatological indications included psoriasis (39.0 %), atopic dermatitis (23.3 %), hidradenitis suppurativa (21.2 %), vitiligo (9.0 %), and skin cancer (7.6 %). A significant proportion of the participants (65 %) had been living with their dermatological condition for over 10 years, with an average duration of 23.9 years (SD 19.5). Only 18 % rated their health as excellent or very good, while 36.6 % rated it as fair or poor. The rate of missing data was below 5 % for all items, except for regional variation (21 missing, 6.1%), education (18 missing, 5.2%), and time since the first diagnosis (18 missing, 5.2%).

**TABLE 1:** Sociodemographic characteristics of the total study population and identified clusters.

	Total Study Population^*^ (n = 344)	Digital Sceptics (Cluster 1) (n = 49)	Cautious Adopters (Cluster 2) (n = 106)	Digital Enthusiasts (Cluster 3) (n = 98)	Adopters Unsure About Impact (Cluster 4) (n = 62)	p
*Age in years, n (%)*						< 0.001
18–44	105 (31.5)	9 (18.4)	38 (35.8)	42 (44.7)	14 (23.3)	
45–64	147 (44.1)	18 (36.7)	49 (46.2)	43 (45.7)	29 (48.3)	
65+	81 (24.3)	22 (44.9)	19 (17.9)	9 (9.6)	17 (28.3)	
Missing	11	0	0	4	2	
Mean (SD)	52.5 (14.8)	59.2 (13.0)^b^, ^c^	49.8 (14.5)^a^	47.1 (13.8)^a^, ^d^	55.5 (13.6)^c^	< 0.001
*Sex, n (%)*						0.45
Female	247 (74.0)	35 (72.9)	84 (80.0)	68 (71.6)	43 (70.5)	
Male	87 (26.0)	13 (27.1)	21 (20.0)	27 (28.4)	18 (29.5)	
Missing	10	1	1	3	2	
*Regional variation, n (%)*						
Urban or city	245 (75.9)	38 (82.6)	77 (74.8)	66 (72.5)	46 (75.7)	0.62
Rural county	78 (24.1)	8 (17.4)	26 (25.2)	25 (27.5)	14 (23.3)	
Missing, n	21	3	3	7	2	
*Education, n (%)*						< 0.001
Low	28 (8.1)	6 (12.5)	11 (10.9)	8 (8.2)	1 (1.8)	
Medium	106 (30.8)	20 (41.7)	43 (42.6)	21 (21.4)	15 (26.3)	
High	192 (58.9)	22 (45.8)	47 (46.5)	69 (70.4)	41 (71.9)	
Missing	18	1	5	0	5	
*Indication, n (%)*						
Psoriasis	134 (39.0)	23 (46.9)	37 (34.9)	26 (26.5)	31 (50.0)	0.010
Atopic dermatitis	80 (23.3)	16 (32.7)	23 (21.7)	22 (22.4)	13 (21.0)	0.43
Vitiligo	31 (9.0)	4 (8.2)	10 (9.4)	9 (9.2)	5 (8.1)	0.98
Skin cancer	26 (7.6)	2 (4.1)	8 (7.5)	12 (12.2)	3 (4.8)	0.23
Hidradenitis suppurativa	73 (21.2)	4 (8.2)	27 (25.5)	29 (29.6)	12 (19.4)	0.025
*Time since the first diagnosis in years, n (%)*						0.010
0–4	66 (21.8)	4 (8.9)	24 (24.2)	30 (30.9)	8 (12.9)	
5–9	40 (13.2)	3 (6.7)	15 (15.2)	14 (14.4)	8 (12.9)	
10+	197 (65.0)	38 (84.4)	60 (60.6)	53 (54.6)	46 (74.2)	
Missing	18	4	7	1	0	
Mean, SD	23.9 (19.5)	34.7 (21.2)^b^, ^c^	23.0 (19.7)^a^	17.8 (17.1)^a^, ^d^	26.9 (17.6)^c^	< 0.001
*General health perception (SF‐36; 1 Item), n (%)*						0.20
Excellent or Very good	62 (18.0)	5 (10.2)	16 (15.1)	24 (24.5)	14 (22.6)	
Good	152 (44.2)	23 (46.9)	48 (45.3)	35 (35.7)	30 (48.8)	
Fair or poor	126 (36.6)	21 (42.9)	42 (39.6)	39 (39.8)	18 (29.0)	
Missing	4	0	0	0	0	

*Abbr*.: SD, standard deviation; SF‐36, Short Form Health Survey‐36

^a^ Different from Cluster 1 (Digital Sceptics), p < 0.05

^b^ Different from Cluster 2 (Cautious Adopters), p < 0.05

^c^ Different from Cluster 3 (Digital Enthusiasts), p < 0.05

^d^ Different from Cluster 4 (Adopters Unsure About Impact), p < 0.05

* 29 participants could not be used in the cluster analysis because of missing values of the mean scores.

### Factors of acceptability

The suitability of the data for conducting an EFA was confirmed by the KMO criterion (0.90) and Bartlett's Test of Sphericity (X^2^ (df: 171) = 3335, p < 0.001). Four factors with eigenvalues greater than 1 were identified (Online Supplement S3), with calculated Cronbach's Alpha values (0.89, 0.82, 0.79, and 0.91) indicating at least acceptable internal consistency. The following factor names were assigned:

- Factor 1: “Acceptability of DHIs and Trust” comprised ten items,
- Factor 2: “Patients’ Digital Health Competencies” comprised six items,
- Factor 3: “Digital Health Insecurities” comprised five items,
- Factor 4: “Positive Impact of DHIs” comprised five items.

Mean scores could range from 1 to 5, where higher scores indicated a higher agreement with the underlying items. In factors 1, 2, and 4, higher scores reflect more positive perceptions, whereas in factor 3, higher scores reflect more negative perceptions, in line with the connotations of the underlying items. The mean scores for factor 1 and factor 4 were moderate to high (Factor 1: M 3.9, SD 0.7; Factor 4: M 3.6, SD 1.0), while the mean score for factor 2 was high (M 4.0, SD 0.7), and the one for factor 3 was low (M 2.4, SD 0.4) (Table 2).

**TABLE 2:** Differences in identified subscales between clusters.

	Total study population (n = 344)^*^	Digital Sceptics (Cluster 1) (n = 49)	Cautious Adopters (Cluster 2) (n = 106)	Digital Enthusiasts (Cluster 3) (n = 98)	Adopters Unsure About Impact (Cluster 4) (n = 62)	p value (ANOVA)
Mean, standard deviation						
Factor 1: Acceptability of DHIs and trust	3.9 (0.7)	2.8 (0.6)^b^, ^c^, ^d^	3.9 (0.4)^a^, ^c^	4.5 (0.3)^a^, ^b^, ^d^	3.9 (0.6) ^a^, ^c^	< 0.001
Factor 2: Patients’ digital health competencies	4.0 (0.7)	3.2 (0.7)^b^, ^c^, ^d^	3.8 (0.5)^a^, ^c^, ^d^	4.5 (0.4)^a^, ^b^	4.3 (0.5)^a^, ^b^	< 0.001
Factor 3: digital health insecurities	2.4 (0.4)	3.0 (0.6)^c^, ^d^	2.9 (0.5)^c^, ^d^	1.8 (0.5)^a^, ^b^	1.7 (0.4)^a^, ^b^	< 0.001
Factor 4: Positive impact of DHIs	3.6 (1.0)	2.2 (0.6)^b^, ^c^, ^d^	3.8 (0.5)^a^, ^c^, ^d^	4.5 (0.5)^a^, ^b^, ^d^	2.8 (0.6)^a^, ^b^, ^c^	< 0.001

^a^ Different from Cluster 1 (Digital Sceptics), p < 0.05

^b^ Different from Cluster 2 (Cautious Adopters), p < 0.05

^c^ Different from Cluster 3 (Digital Enthusiasts), p < 0.05

^d^ Different from Cluster 4 (Adopters Unsure About Impact), p < 0.05

* 29 participants could not be used in the cluster analysis because of missing values of the mean scores.

### Clusters of patients based on acceptability levels

The optimal solution, determined through hierarchical cluster analysis, resulted in a four‐cluster classification. Subsequent k‐means (ANOVA and post hoc tests) confirmed significant variations across all four subscales (Table 2).

Cluster 1 (n = 49; 15.6 %) demonstrated moderate ratings for acceptability and trust (Factor 1; M 2.8, SD 0.6) and competencies (Factor 2: M 3.2, SD 0.7), along with low expectancies for a positive impact of DHIs (Factor 4: M 2.2, SD 0.6). All factors were rated significantly lower compared with the other three clusters. Additionally, digital insecurities were moderate (Factor 3: M 3.0, SD 0.6) and significantly higher than those in clusters 3 and 4.

Cluster 2 (n = 106; 33.7 %) showed moderate to high mean scores in acceptability and trust (Factor 1: M 3.9, SD 0.5), competencies (Factor 2: M 3.8, SD 0.5), and expectancies of a positive impact (Factor 4: M 3.8, SD 0.5). Acceptability and trust were significantly lower than in Cluster 3, and competencies and positive expectancies were lower compared to clusters 3 and 4. Digital insecurities (Factor 3: M 2.9, SD 0.5) were moderate and significantly higher than those in clusters 3 and 4.

Cluster 3 (n = 98; 31.1 %) displayed the highest ratings on acceptability and trust (Factor 1: M 4.5, SD 0.3) as well as expectancies of a positive impact of DHIs (Factor 4: M 4.5, SD 0.5) among all clusters. Digital competencies were also rated high (Factor 2: M 4.5; SD 0.4), whereas digital insecurities were low (Factor 3: M 1.8, SD 0.5). Both mean scores were significantly higher than those of clusters 1 and 2.

Cluster 4 (n = 62; 18.0 %) was similar to cluster 2 in terms of acceptability of DHIs and trust (Factor 1: M 3.9; SD 0.6), and to cluster 3 in terms of competencies and insecurities (Factor 2: M 4.3, SD 0.5; Factor 3: M 1.7, SD 0.5). However, cluster 4 (Factor 4: M 2.8, SD 0.6) had a significantly lower perception of the potential positive impact of DHIs than clusters 2 (M 3.8, SD 0.5) and 3 (M 4.5, SD 0.5).

For clarity, we refer to clusters 1, 2, 3, and 4 as “Digital Sceptics”, “Cautious Adopters”, “Digital Enthusiasts”, and “Adopters Unsure About Impact” in subsequent sections.

### Sociodemographic and health‐specific differences of clusters

In terms of sociodemographic parameters, the cluster analysis revealed significant differences among the identified groups (Table 1). “Digital Enthusiasts” (M 47.1 years; SD 13.8) were notably younger than both “Digital Sceptics” (M 59.2 years; SD 13.0) and “Adopters Unsure About Impact” (M 55.5 years; SD 13.6). “Cautious Adopters” were younger compared to “Digital Sceptics” (M 49.8; SD 14.5 vs. 59.2 years; SD 13.0). No statistically significant sex or geographical variations were observed. A significant association was noted in the educational level with “Digital Enthusiasts” and “Adopters Unsure About Impact” having a higher level of education (70.4 % and 71.9 %) compared with “Digital Sceptics” and “Cautious Adopters” (45.8 % and 46.5 %).

A significant association was identified between the cluster assignment of psoriasis and hidradenitis suppurativa. Participants diagnosed with psoriasis were more prevalent in the “Digital Sceptics” (46.9 %) and “Adopters Unsure About Impact” (50.0 %) clusters, whereas those diagnosed with hidradenitis suppurativa had a larger proportion in the “Cautious Adopters” (25.5 %) and “Digital Enthusiasts” (29.6 %) clusters. The time since first diagnosis varied significantly among the four clusters. Specifically, “Digital Sceptics” had a significantly longer time since first diagnosis (M 34.7 years; SD 21.2) compared to “Cautious Adopters” (M 23.0 years; SD 19.7) and “Digital Enthusiasts” (M 17.8 years; SD 17.1) (Table 1). In addition, “Adopters Unsure About Impact” had a significantly longer time since diagnosis than “Digital Enthusiasts” (M 26.9 years; SD 16.6 vs. 17.8; SD 17.1).

### Acceptability of DHIs within identified clusters

**FIGURE 1:**
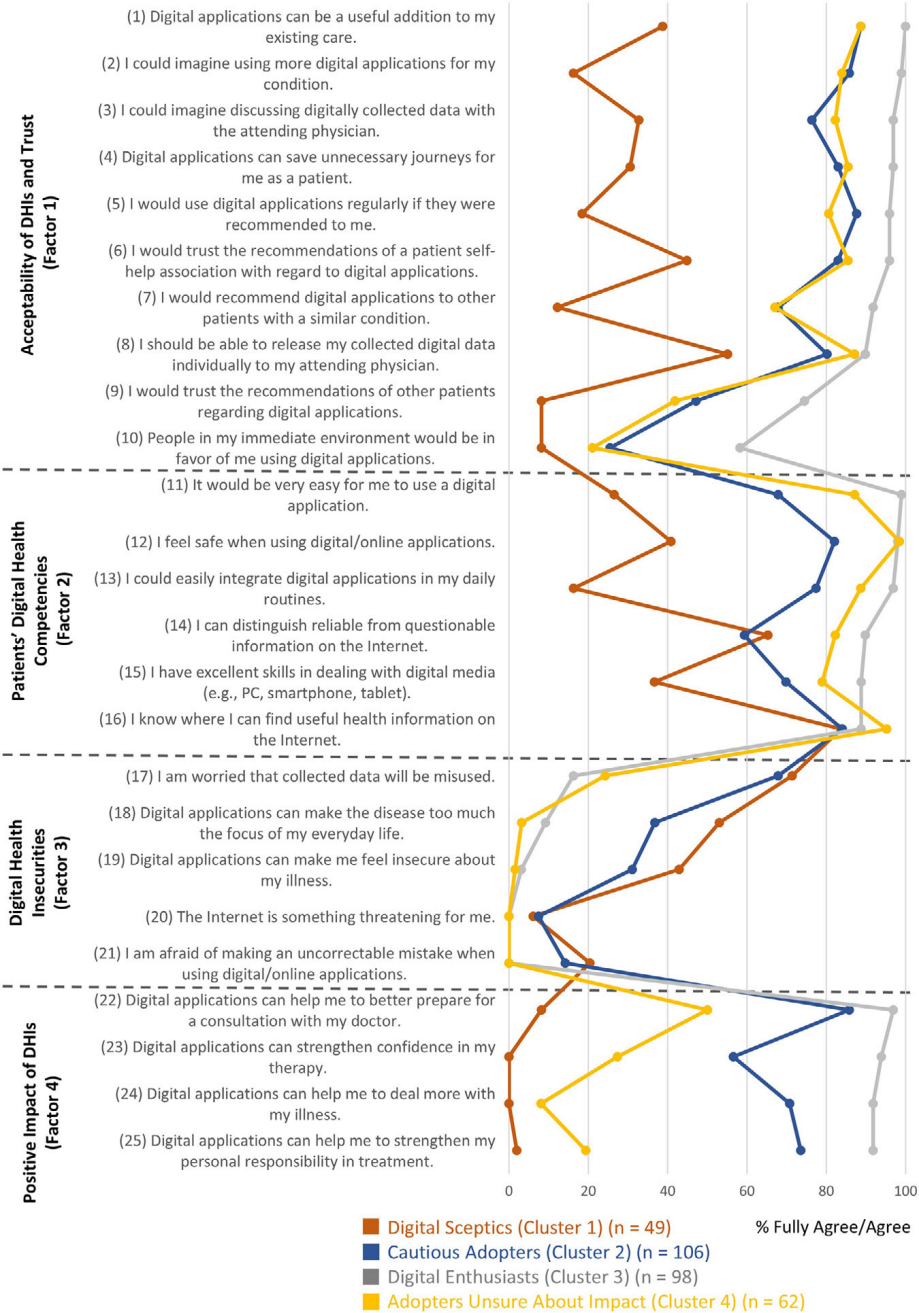
Cluster profile analysis at the item‐by‐item level.

The subsequent paragraphs offer a detailed description of items revealing unique profiles for each of the four clusters in their acceptability towards DHIs. A comprehensive summary of the ratings for all items is presented in Figure 1. An overview of the overall group (N = 344) is displayed within the supplemental material (Online Supplement S5; Table S7).

“Digital Sceptics” demonstrated low agreement rates on items within factor 1, ranging from 8.2 % (Item 9: trust in recommendations of other patients) to 55.1 % (Item 8: release of data to my attending physician) (Figure 1; Factor 1). Regarding factor 2, participants self‐reported low digital skills (Item 11: 26.5 %) but also proficiency in finding useful online information (Item 16: 83.7 %) and the ability to distinguish reliable from questionable information (Item 14: 65.3 %) (Figure 1; Factor 2). “Digital Sceptics” expressed concerns about the potential misuse of their data (Item 17: 71.3 %) (Figure 1; Factor 3). However, the group did not feel intimated by the internet (Item 28: 14.2 %) (Figure 1; Factor 3). Ratings on the positive impacts of DHIs were low, with no one in the cluster expecting DHIs to enhance confidence in therapy for their disease (Item 23: 0.0 %) (Figure 1; Factor 4).

The “Cautious Adopters” and “Adopters Unsure About Impact” displayed high ratings in factor 1. Both believed that applications could be a useful addition to existing care (Item 1; “Cautious Adopters”: 88.7 %; “Adopters Unsure About Impact”: 88.7 %) but were uncertain about support from their immediate environment to use DHIs (Item 11: “Cautious Adopters:” 25.5 %, “Adopters Unsure About Impact”: 21.0 %). Among “Adopters Unsure About Impact”, ratings on competency items in factor 2 (Figure 1) were high, while the “Cautious Adopters” had lower agreement rates with items in this factor. For example, 59.4 % of “Cautious Adopters” considered themselves able to distinguish reliable from questionable data (Item 14), whereas 82.3 % agreed among the “Adopters Unsure About Impact” (Figure 1; Factor 2). In factor 3, the “Cautious Adopters” expressed significant concerns about potential data misuse (67.9 %; Item 17), compared to the “Adopters Unsure About Impact” (22.3 %). Overall, this latter group showed low agreement rates across factor 3. For example, only 1.6 % agreed with the statement that DHIs could make them feel insecure about their disease (Item 19). In contrast, regarding the positive impact of DHIs (Figure 1; Factor 4), “Cautious Adopters” revealed higher agreement rates, ranging from 56.6 % (Item 23: strengthening confidence in therapy) to 85.8 % (Item 22: better preparation for consultations with their doctor). In comparison, “Adopters Unsure About Impact” reported lower rates, varying from 8.1 % (Item 24: support in managing their disease) to 50.0 % (Item 22: strengthening confidence in therapy).

“Digital Enthusiasts” showed high acceptability ratings in all factors (Figure 1). Agreement rates for most items in factors 1, 2, and 4 ranged from 85 % to 100 %. An exception was noted in factor 1, where 74.5 % trusted recommendations from other patients (Item 9), and 58.2 % believed that people in their environment would support DHIs (Item 11). Ratings on items in “Digital Health Insecurities” (Figure 1; Factor 3) were generally low; e.g., only a minority expressed concerns about possible data misuse (Item 17: 16.3 %).

Overall, the majority of participants across all clusters agreed that they should be able to grant their attending physician access to read their data (Item 8; Factor 1; Range: 55.1 %–89.9 %) and that they are aware of where to find useful health information online (Item 16; Factor 3; Range: 83.7 %–95.2 %).

### Usage of DHIs within identified clusters

**FIGURE 2:**
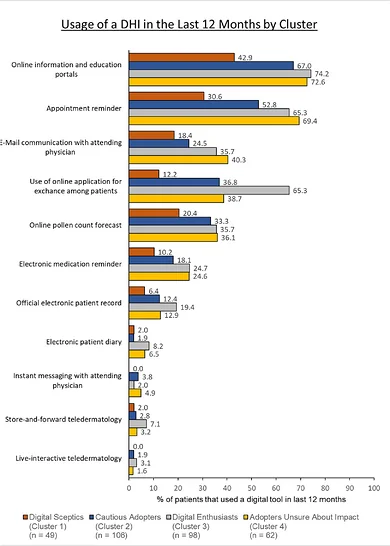
Use of a DHI in the last 12 months by cluster.

Overall, the adoption of DHIs was limited, with only four DHIs – online pollen forecast (31.4 %), online exchange (40.1 %), appointment reminders (53.5 %), and educational portals (63.7 %) – being used by at least one third of the overall group. Particularly low utilization rates were observed for live‐interactive teledermatology (2.1 %) and store‐and‐forward teledermatology (4.1 %) (Supplement S5; Table S8). “Digital Sceptics” consistently demonstrated low usage rates across all DHIs. In contrast, both “Adopters Unsure About Impact” and “Digital Enthusiasts” had the highest usage rates (Figure 2). For example, 72.6 % (“Adopters Unsure About Impact”) and 74.2 % (“Digital Enthusiasts”) used online information portals. “Cautious Adopters” showed comparable rates to “Adopters Unsure About Impact” in certain DHIs, such as online applications for patient exchange (38.7 % vs. 36.8 %), but markedly lower adoption in others, such as email communication with treating physicians (40.3 % vs. 24.5 %). DHIs that facilitate communication with physicians, excluding e‐mail, were generally infrequently used. For instance, store‐and‐forward (S&F) teledermatology was used by 7.1 % of “Digital Enthusiasts” and 2.0 % of “Digital Sceptics.” The official ePA also demonstrated low usage across all clusters, ranging from 6.4 % in “Digital Sceptics” to 19.4 % in “Digital Enthusiasts.”

### Potential future usage of DHIs within identified clusters

**FIGURE 3:**
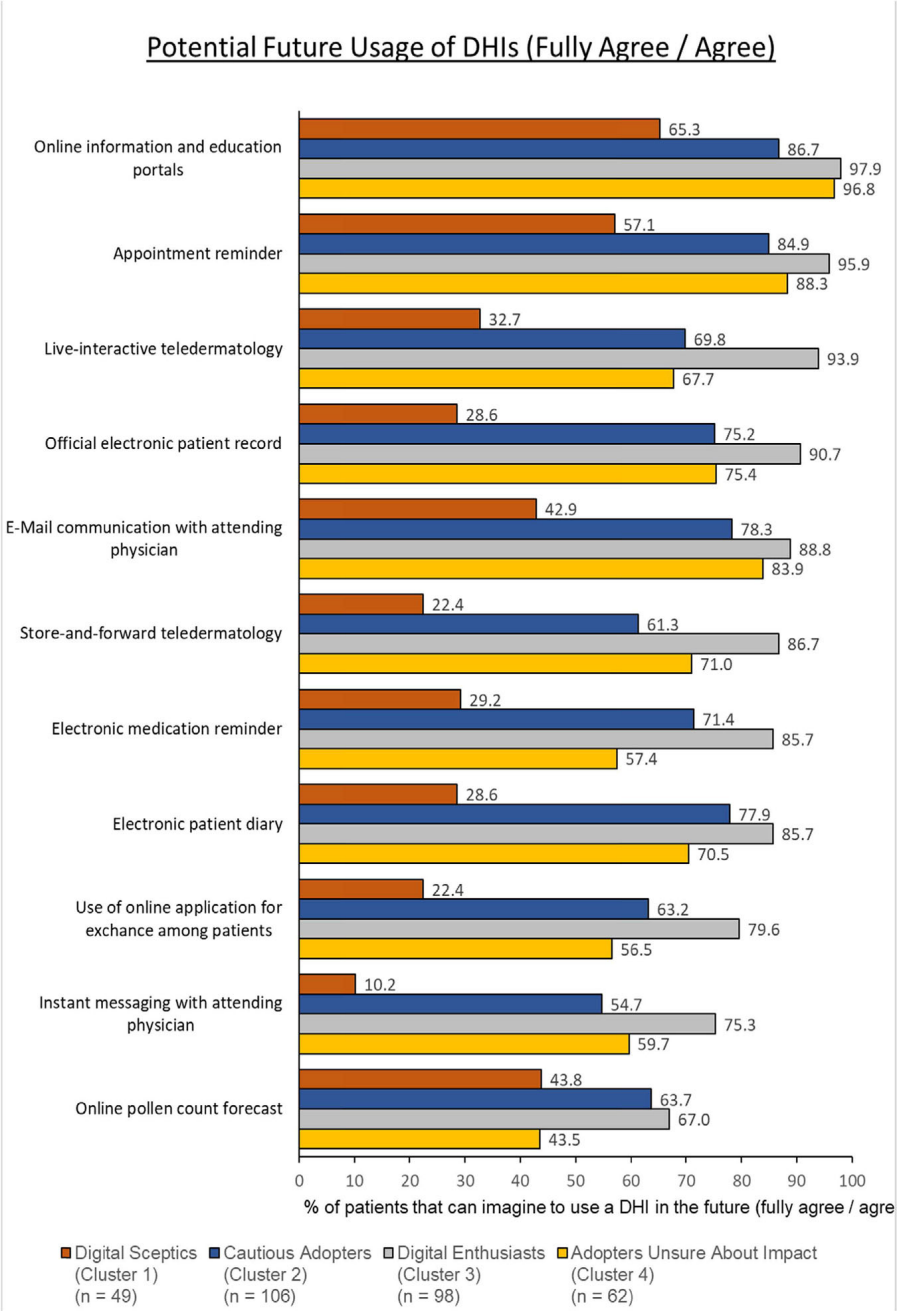
Potential future use of DHIs.

For potential future use, at least 50 % of patients reported they would consider using each of the eleven DHIs more frequently, ranging from 52.3 % for instant messaging with attending physicians to 85.5 % for online information and education portals (Online Supplement S5; Table S9). When considering only “Digital Sceptics” (Cluster 1) only about one‐third showed interest in using seven out of the eleven DHIs more frequently. Exceptions included education portals (65.3 %), appointment reminders (57.1 %), the online pollen count forecast (43.8 %), and email communications (42.9 %). In contrast, the clear majority within the three adopters’ clusters (Clusters 2–4) expressed a willingness to more often use DHIs (Figure 3). For five out of the eleven presented DHIs, at least two thirds within each adopter's cluster indicated an openness to increased usage. The DHIs with the highest agreement rates among adopters’ clusters were online information and educational portals (“Cautious Adopters:” 86.7 % to “Digital Enthusiasts:” 97.9 %), appointment reminders (“Cautious Adopters:” 84.9 % to “Digital Enthusiasts”: 95.9 %), and the ePA (“Cautious Adopters:” 75.2 % to 90.7 %). Within the “Digital Enthusiasts,” rates for ten out of the eleven presented DHIs consistently exceeded 75 %, reaching as high as 97.9 % for the potential future usage of education portals for their disease.

## DISCUSSION

The objective of this study was to investigate the level of acceptability as well as the current and potential future usage of DHIs among patients with skin diseases. Further, clusters were identified according to their level of acceptability as well as their associations between sociodemographic characteristics, current, and future usage of DHIs. A majority of patients and three out of four identified clusters (“Cautious Adopters”, “Digital Enthusiasts”, “Adopters Unsure About Impact”) expressed a willingness to embrace DHIs more often in the future. Furthermore, the cluster “Digital Enthusiasts” possessed sufficient digital competencies and expressed low levels of digital health insecurities. The “Cautious Adopters” showed a positive attitude toward DHIs but expressed concerns about data misuse. The “Adopters Unsure About Impact” did not expect any positive effects of DHIs on their disease handling. The other cluster, the “Digital Sceptics”, demonstrated a general unwillingness to use DHIs, had limited digital competencies and a low perceived benefit of these interventions. Even among patients who were open to adopt DHIs, the current utilization rates for existing interventions, such as teledermatology, ePA, and electronic patient diaries, remained relatively modest.

Our findings emphasize a high interest of patients with skin diseases in using DHIs, but for example, only 7.1 % of the most digitally minded cluster have already used S&F teledermatology. There may be systemic barriers, such as the limited availability of services. So even at the height of the COVID‐19 pandemic, only a minority of dermatologists in Germany (21 %) offered S&F teledermatology to their patients.^19^, ^20^ In addition, S&F teledermatology solutions are currently not designed for chronic patients but focus on the initial assessment of patients.^15^, ^35^, ^36^ In regards to other DHIs, only a minority of DHIs discovered in the medical literature were designed specifically for patients with skin diseases,^11^ and only 2 % of disease‐specific health apps were designed for dermatological purposes.^37^

Interestingly, many physicians attribute their reluctance to introduce DHIs to a perceived lack of interest among patients.^38^, ^39^ Our data revealed that many patients with chronic dermatological diseases would endorse using these services. One possible explanation for this discrepancy may be the insufficient integration of current DHIs into existing care, leading patients and physicians to prefer the standard of care. However, DHIs capable of ensuring continuity of care while reducing waiting times and integrating seamlessly with current care are more likely to be preferred by patients.^40^, ^41^

Digital insecurities, especially regarding data misuse, emerged as another significant barrier for the “Cautious Adopters” and “Digital Sceptics”. This negative association was already identified in previous research.^42^, ^43^, ^44^ Enhancing the uptake of DHIs necessitates not only ensuring data security and privacy but also actively informing patients about the security measures and use of their data.

The discovery of a patient cluster with a high intention to use DHIs (“Adopters Unsure About Impact”), possessing sufficient digital skills and having low digital insecurities, but still expecting low impact of DHIs on their care is interesting. Performance expectancy, in our study titled “Positive Impact of DHIs”, is often the most robust predictor of DHI acceptability.^27^ This cluster, with 74 % of patients having their skin condition for over 10 years, may already have adapted to the demands of their chronic skin conditions, resulting in lower expectations towards the additional value offered by DHIs.

Our research identified a second‐order digital divide characterized by disparities in digital competencies, acceptability, and usage instead of infrastructure and access devices (e.g. smartphones and computers).^45^ In alignment with previous studies,^46^, ^47^ our findings underscored that advanced age and lower education were associated with lower use of DHIs. This finding is concerning because access to and usage of digital health services is an increasingly important determinant of health, especially since the COVID‐19 Pandemic.^46^, ^48^ Some researchers even proposed a comprehensive framework for Digital Health Equity.^49^ Potential actions include involving patients of different ages and education levels within the development of DHIs, ensuring the usability of DHIs for marginalized populations (e.g. simplified app modes), educating health care professionals, and providing digital literacy programs within local communities, such as through libraries.^48^, ^49^ Health insurers could support such efforts as they already have a responsibility to promote DHIs and enhance digital literacy to ensure their appropriate use.^50^ Further data are essential to better understand the relationship between digital inequities and health disparities, enabling the development of effective countermeasures.

The cross‐sectional survey employed has several limitations. Patients were invited to participate through patient organizations. Based on research, this patient group may experience a greater disease burden, have higher educational levels, receive more information, and may possess a more comprehensive understanding of their disease.^51^ Thus, they may not be representative of the broader population affected by chronic skin conditions. The study also focused on persons with chronic skin diseases of rather high severity. Therefore, the results may not capture acute or mild skin diseases. Furthermore, within our study population more women and older participants took part in comparison to the general population with skin diseases, also observable for each of the assessed indications (compare Online Supplement S4). There may be a selection bias, as patients with greater digital affinity – particularly due to recruitment via email and social media – were more likely to respond to the survey. Taking all aspects into account, the actual size of the different clusters may vary in the general skin‐disease population.

Although a relationship between clusters and adoption rates has been identified, e.g. high adoption by “Digital Enthusiasts” and low by “Digital Sceptics,” this cannot be projected into the future. First, the cross‐sectional design prevents the establishment of the direction of the identified relationship. Second, the survey is susceptible to the intention–behavior gap.^52^ Therefore, even if acceptability drives adoption, the intention to adopt DHIs in the future may not necessarily translate into actual uptake.

Exploratory factor analysis was chosen instead of confirmatory factor analysis for data reduction because no theoretical assumptions about item interactions were made. The exploratory approach limits comparability with other research on acceptability, which often uses the UTAUT framework as a methodological foundation.^27^, ^53^ Another limitation is that, as recommended, no confirmatory factor analysis was performed because the sample size did not allow for splitting the dataset.^54^ Despite this limitation, EFA was deemed appropriate based on the KMO criterion and Bartlett's test of sphericity, yielding factors with acceptable internal consistency.^34^

## CONCLUSIONS

The results of our survey revealed that patients with chronic skin conditions can be attributed to four different types of digital users. Most patients within the identified clusters are willing to use DHIs more often in the future. We found high acceptability. However, the current use of interventions is low to moderate, likely because of several barriers, including the limited availability of such DHIs that are inadequately implemented into or reimbursed by the health system. Data privacy concerns and limited digital competencies among patients should be acknowledged by developers, researchers, and even health insurers to improve the uptake of DHIs and reduce differences in access to DHIs that may lead to digital health inequities. Patients belonging to the more skeptical group of digital users deserve special attention in practice, including more empowerment and shared decisions before use of digital technology. Moreover, the acceptance and skills of physicians in dealing with DHIs should be increased as their opinion may create a barrier, as well. Ongoing research and collaborative efforts are essential to enhance the adoption and inclusivity of digital health solutions in dermatology.

## FUNDING

This research project was financially supported by a research grant from Novartis Pharma GmbH. Novartis did not influence the design, data collection, analysis, publication decision, or development of this study.

## CONFLICT OF INTEREST STATEMENT

M.O. is a co‐author of the German AWMF guideline on teledermatology. M.A. is a co‐author of the German AWMF guideline on teledermatology and serves as a scientific advisor for the teledermatology platforms derma2go AG, A+ Videoclinic GmbH, and Novartis Pharma GmbH. P.R. declares no conflict of interest.

## Supporting information



Supplementary information

Supplementary information

Supplementary information

Supplementary information

## References

[1] Svensson A , Ofenloch RF , Bruze M , et al. Prevalence of skin disease in a population‐based sample of adults from five European countries. Br J Dermatol. 2018;178:1111‐1118. 29247509 10.1111/bjd.16248

[2] Cribier B , Aroman MS , Merhand S , et al. Prevalence of visible skin diseases: An international study of 13,138 people. J Eur Acad Dermatol Venereol. 2023;37:e180‐e182. 36251446 10.1111/jdv.18666

[3] Augustin M , Herberger K , Hintzen S , et al. Prevalence of skin lesions and need for treatment in a cohort of 90 880 workers. Br J Dermatol. 2011;165:865‐873. 21623753 10.1111/j.1365-2133.2011.10436.x

[4] Pilz AC , Zink A , Schielein MC , et al. Despite large choice of effective therapies: Individuals with psoriasis still seem undertreated. J Dtsch Dermatol Ges. 2021;19:1003‐1011. 10.1111/ddg.14387 33955676

[5] Kis A , Augustin M , Augustin J . Regional healthcare delivery and demographic change in Germany ‐ scenarios for dermatological care in 2035. J Dtsch Dermatol Ges. 2017;15:1199‐1209. 10.1111/ddg.13379 29228491

[6] van der Kraaij GE , Vermeulen FM , Smeets PMG , et al. The current extent of and need for shared decision making in atopic dermatitis and psoriasis in the Netherlands: an online survey study amongst patients and physicians. J Eur Acad Dermatol Venereol. 2020;34:2574‐2583. 32163645 10.1111/jdv.16340 PMC7818257

[7] Augustin M , Girbig G , Kis A , et al. Inpatient care for skin diseases in Germany: multi‐source analysis on the current and future health care needs. J Dtsch Dermatol Ges. 2021;19(Suppl 5):25‐53. 10.1111/ddg.14620 34662491

[8] Wildenbos GA , Peute LW , Jaspers MWM . Impact of Patient‐centered eHealth Applications on Patient Outcomes: A Review on the Mediating Influence of Human Factor Issues. Yearb Med Inform. 2016;25(1):113‐119. 10.15265/IY-2016-031 PMC5171552 27830238

[9] Eysenbach G . What is e‐health? J Med Internet Res. 2001;3:E20. 11720962 10.2196/jmir.3.2.e20 PMC1761894

[10] Boogerd EA , Arts T , Engelen LJ , et al. “What Is eHealth”: Time for An Update? JMIR Res Protoc. 2015;4:e29. 25768939 10.2196/resprot.4065 PMC4376129

[11] Reinders P , Augustin M , Kirsten N , et al. Digital health interventions in dermatology‐Mapping technology and study parameters of systematically identified publications. J Eur Acad Dermatol Venereol. 2023;37:2440‐2449. 37528462 10.1111/jdv.19392

[12] Liu Y , Jain A , Eng C , et al. A deep learning system for differential diagnosis of skin diseases. Nat Med. 2020;26:900‐908. 32424212 10.1038/s41591-020-0842-3

[13] Armstrong AW , Ford AR , Chambers CJ , et al. Online Care Versus In‐Person Care for Improving Quality of Life in Psoriasis: A Randomized Controlled Equivalency Trial. J Invest Dermatol. 2019;139:1037‐1044. 30481495 10.1016/j.jid.2018.09.039 PMC6599714

[14] Koch R , Rösel I , Polanc A , et al. TELEDerm: Implementing store‐and‐forward teledermatology consultations in general practice: Results of a cluster randomized trial. J Telemed Telecare. 2022;30(4):647‐660. 35578544 10.1177/1357633X221089133

[15] Abeck F , Kött J , Bertlich M , et al. Direct‐to‐Consumer Teledermatology in Germany: A Retrospective Analysis of 1,999 Teleconsultations Suggests Positive Impact on Patient Care. Telemed J E Health. 2023;29(10):1484‐1491. 36862525 10.1089/tmj.2022.0472

[16] Svendsen MT , Andersen F , Andersen KH , et al. A smartphone application supporting patients with psoriasis improves adherence to topical treatment: a randomized controlled trial. Br J Dermatol. 2018;179:1062‐1071. 29654699 10.1111/bjd.16667

[17] Augustin M , Strömer K , et al. S2k‐Leitlinie Teledermatologie, 2020 Available from: https://www.awmf.org/leitlinien/detail/ll/013‐097.html [Last accessed May 25, 2022].

[18] Thiel R , Deimel L , Schmidtmann D , et al. #SmartHealthSystems. Digitalisierungsstrategien im internationalen Vergleich. Bertelsmann Stiftung (Hrsg.), 2018. Available from: https://www.bertelsmann‐stiftung.de/de/publikationen/publikation/did/smarthealthsystems [Last accessed August 15, 2025].

[19] Augustin M , Reinders P , Janke TM , et al. Attitudes Toward and Use of eHealth Technologies Among German Dermatologists: Repeated Cross‐Sectional Survey in 2019 and 2021. J Med Internet Res. 2024;26:e45817. 38345855 10.2196/45817 PMC10897787

[20] Elsner P . Teledermatology in the times of COVID‐19 ‐ a systematic review. J Dtsch Dermatol Ges. 2020;18:841‐845. 10.1111/ddg.14180 33448667

[21] Reinders P , Augustin M , Otten M . General and dermatological population's use and acceptance of digital health in Germany ‐ a representative survey. J Dtsch Dermatol Ges. 2024;22(9):1221‐1231. 39555644 10.1111/ddg.15454

[22] Nohl‐Deryk P , Brinkmann JK , Gerlach FM , et al. Hürden bei der Digitalisierung der Medizin in Deutschland – eine Expertenbefragung. Gesundheitswesen. 2018;80:939‐945. 29301149 10.1055/s-0043-121010

[23] Reinders P , Augustin M , Fleyder A , et al. Exploring Acceptability, Barriers, and Facilitators for Digital Health in Dermatology: Qualitative Focus Groups with Dermatologists, Nurses, and Patients (Preprint). JMIR Dermatology. 2024;3:7:e57172. 10.2196/57172 PMC11408893 39226097

[24] Klein TM , Augustin M , Kirsten N , et al. Attitudes towards using electronic health records of patients with psoriasis and dermatologists: a cross‐sectional study. BMC Med Inform Decis Mak. 2020;20(1):344. 33380329 10.1186/s12911-020-01302-y PMC7772927

[25] Greis C , Meier Zürcher C , Djamei V , et al. Unmet digital health service needs in dermatology patients. J Dermatolog Treat. 2018;29:643‐647. 29455570 10.1080/09546634.2018.1441488

[26] Sharma A , Minh Duc NT , Luu Lam Thang T , et al. A Consensus‐Based Checklist for Reporting of Survey Studies (CROSS). J Gen Intern Med. 2021;36:3179‐3187. 33886027 10.1007/s11606-021-06737-1 PMC8481359

[27] Philippi P , Baumeister H , Apolinário‐Hagen J , et al. Acceptance towards digital health interventions – Model validation and further development of the Unified Theory of Acceptance and Use of Technology. Internet Interv. 2021;26:100459. 34603973 10.1016/j.invent.2021.100459 PMC8463857

[28] Soellner R , Huber S , Reder M . The Concept of eHealth Literacy and Its Measurement. J Media Psychol. 2014;26(1):29‐38.

[29] Bullinger M , Kirchberger I , Ware J . Der deutsche SF‐36 Health Survey Übersetzung und psychometrische Testung eines krankheitsübergreifenden Instruments zur Erfassung der gesundheitsbezogenen Lebensqualität. J Public Health. 1995;3:21‐36.

[30] Anderson JC , Gerbing DW . Structural equation modeling in practice: A review and recommended two‐step approach. Psychol Bull. 1988;103:411‐423.

[31] World Medical Association Declaration of Helsinki: ethical principles for medical research involving human subjects. JAMA. 2013;310(20):2191‐2194. 24141714 10.1001/jama.2013.281053

[32] Deutsche Forschungsgemeinschaft . Guidelines for Safeguarding Good Research Practice. Code of Conduct. 20219. Available from: 10.5281/zenodo.3923602. (Last accessed August 15, 2025).

[33] Floyd FJ , Widaman KF . Factor analysis in the development and refinement of clinical assessment instruments. Psychol Assess. 1995;7(3):286‐299.

[34] Howard MC . A Review of Exploratory Factor Analysis Decisions and Overview of Current Practices: What We Are Doing and How Can We Improve? Int J Hum‐Comput Interact. 2016;32(1):51‐62.

[35] Otten M , Greis C , Reinders P , et al. Patient and physician outcomes of a store‐and‐forward teledermatology application in Germany. J Eur Acad Dermatol Venereol. 2024;38:e148‐e151. 37669866 10.1111/jdv.19501

[36] Sondermann W , Kalle C von , Utikal JS , et al. Externe wissenschaftliche Evaluation der ersten Teledermatologie‐App ohne direkten Patientenkontakt in Deutschland („Online Hautarzt – AppDoc“). Hautarzt. 2020;71:887‐897. 32728813 10.1007/s00105-020-04660-w PMC7387809

[37] Digital Health Trends 2021: Innovation, Evidence, Regulation, And Adoption. IQVIA Institute. Available from: https://www.iqvia.com/insights/the‐iqvia‐institute/reports‐and‐publications/reports/digital‐health‐trends‐2021. [Last accessed August 15, 2025].

[38] Knörr V , Dini L , Gunkel S , et al. Use of telemedicine in the outpatient sector during the COVID‐19 pandemic: a cross‐sectional survey of German physicians. BMC Prim Care. 2022;23:92. 35461212 10.1186/s12875-022-01699-7 PMC9034069

[39] Witte NAJ de , Carlbring P , Etzelmueller A , et al. Online consultations in mental healthcare during the COVID‐19 outbreak: An international survey study on professionals' motivations and perceived barriers. Internet Interv. 2021;25:100405. 34401365 10.1016/j.invent.2021.100405 PMC8350604

[40] Weinrich P von , Kong Q , Liu Y . Would you zoom with your doctor? A discrete choice experiment to identify patient preferences for video and in‐clinic consultations in German primary care. J Telemed Telecare. 2022;30(6):969‐992. 35915997 10.1177/1357633X221111975

[41] Phillips EA , Himmler SF , Schreyögg J . Preferences for e‐Mental Health Interventions in Germany: A Discrete Choice Experiment. Value Health. 2021;24:421‐430. 33641777 10.1016/j.jval.2020.09.018

[42] Schomakers EM , Lidynia C , Ziefle M . Listen to My Heart? How Privacy Concerns Shape Users’ Acceptance of e‐Health Technologies. *International Conference on Wireless and Mobile Computing, Networking and Communications (WiMob)* . Spain. 2019:306‐311.

[43] Determann D , Lambooij MS , Gyrd‐Hansen D , et al. Personal health records in the Netherlands: potential user preferences quantified by a discrete choice experiment. J Am Med Inform Assoc. 2017;24:529‐536. 28011592 10.1093/jamia/ocw158 PMC7651889

[44] Princi E , Krämer NC . Out of Control – Privacy Calculus and the Effect of Perceived Control and Moral Considerations on the Usage of IoT Healthcare Devices. Front Psychol. 2020;11:582054. 33262731 10.3389/fpsyg.2020.582054 PMC7686240

[45] Elena‐Bucea A , Cruz‐Jesus F , Oliveira T , et al. Assessing the Role of Age, Education, Gender and Income on the Digital Divide: Evidence for the European Union. Inf Syst Front. 2021;23:1007‐1021.

[46] Watts G . COVID‐19 and the digital divide in the UK. Lancet Digit Health. 2020;2:e395‐e396. 32835198 10.1016/S2589-7500(20)30169-2 PMC7384786

[47] Santis KK de , Jahnel T , Sina E , et al. Digitization and Health in Germany: Cross‐sectional Nationwide Survey. JMIR Public Health Surveill. 2021;7:e32951. 34813493 10.2196/32951 PMC8612128

[48] Crawford A , Serhal E . Digital Health Equity and COVID‐19: The Innovation Curve Cannot Reinforce the Social Gradient of Health. J Med Internet Res. 2020;22:e19361. 32452816 10.2196/19361 PMC7268667

[49] Richardson S , Lawrence K , Schoenthaler AM , et al. A framework for digital health equity. NPJ Digit Med. 2022;5:119. 35982146 10.1038/s41746-022-00663-0 PMC9387425

[50] Schaeffer D , Gille S . Gesundheitskompetenz im Zeitalter der Digitalisierung. Präv Gesundheitsf. 2022;17:147‐155. 10.1007/s11553-021-00872-7 PMC8247623 40477284

[51] Langenbruch A , Radtke MA , Foos Z , et al. Benefits of a membership in a psoriasis patient organisation: a quasi‐experimental longitudinal study. Arch Dermatol Res. 2018;310:807‐813. 30350131 10.1007/s00403-018-1869-x

[52] Sheeran P , Webb TL . The Intention‐Behavior Gap. Soc Personal Psychol. 2016;10:503‐518.

[53] Venkatesh , Morris and Davis . User Acceptance of Information Technology: Toward a Unified View. MIS Quarterly. 2003;27:425.

[54] Flora DB , Flake JK . The purpose and practice of exploratory and confirmatory factor analysis in psychological research: Decisions for scale development and validation. Can J Behav Sci. 2017;49:78‐88.

