# Supplementary material for: Attitudes of dermatological patients towards digital health interventions: a cross‐sectional survey and cluster analysis
Source: J Dtsch Dermatol Ges. 2026 Apr 16;24(9):e1264–74. doi: 10.1111/ddg.70010 (PMC13551282; doi:10.1111/ddg.70010)
Supplement: Supplementary file 1 [file DDG-24-e1264-s001.docx]

# **Online Supplement S3 – Results of the Exploratory Factor Analysis**

Table S4 Factor Loadings of the Exploratory Factor Analysis

| Item | Text | Loading | Missing |
| --- | --- | --- | --- |
| Factor 1: Acceptability of DHIs and Trust” (Alpha: 0.89) | | |  |
| 1 | I could imagine using more digital applications for my condition. | 0.805 | 6 (1.7%) |
| 3 | I would recommend digital applications to other patients with a similar condition. | 0.746 | 7 (2.0%) |
| 2 | I would use digital applications regularly if they were recommended to me. | 0.709 | 4 (1.2%) |
| 13 | I could imagine discussing digitally collected data with the attending physician | 0.654 | 3 (0.9%) |
| 14 | I should be able to release my collected digital data individually to my attending physician. | 0.642 | 6 (1.7%) |
| 18 | Digital applications can save unnecessary journeys for me as a patient. | 0.489 | 7 (2.0%) |
| 11 | People in my immediate environment would be in favor of me using digital applications. | 0.470 | 6 (1.7%) |
| 19 | Digital applications can be a useful addition to my existing care. | 0.443 | 6 (1.7%) |
| 10 | I would trust the recommendations of a patient self-help association with regard to digital applications. | 0.428 | 6 (1.7%) |
| 9 | I would trust the recommendations of other patients regarding digital applications. | 0.413 | 5 (1.5%) |
| Factor 2: Patients’ Digital Health Competencies (Alpha: 0.82)* | | | |
| 26 | I have excellent skills in dealing with digital media (e.g., PC, smartphone, tablet). | -0.662 | 8 (2.3%) |
| 27 | I feel safe when using digital/online applications. | -0.657 | 7 (2.0%) |
| 5 | It would be very easy for me to use a digital application. | -0.569 | 5 (1.5%) |
| 31 | I can distinguish reliable from questionable information on the Internet. | -0.507 | 6 (1.7%) |
| 6 | I could easily integrate digital applications in my daily routines. | -0.408 | 6 (1.7%) |
| 30 | I know where I can find useful health information on the Internet. | -0.404 | 5 (1.5%) |
| Factor 3: Digital Health Insecurities (Alpha: 0.79) | | | |
| 16 | Digital applications can make me feel insecure about my illness. | 0.832 | 8 (2.3%) |
| 17 | Digital applications can make the disease too much the focus of my everyday life. | 0.671 | 4 (1.2%) |
| 15 | I am worried that collected data will be misused. | 0.575 | 6 (1.7%) |
| 28 | The Internet is something threatening for me. | 0.532 | 11 (3.2%) |
| 29 | I am afraid of making an uncorrectable mistake when using digital/online applications. | 0.466 | 9 (2.6%) |
| Factor 4: Positive Impact of DHIs (Alpha: 0.91) | | | |
| 21 | Digital applications can help me to deal more with my illness | 0.901 | 8 (2.3%) |
| 22 | Digital applications can help me to strengthen my personal responsibility in treatment. | 0.900 | 9 (2.6%) |
| 20 | Digital applications can strengthen confidence in my therapy. | 0.714 | 9 (2.6%) |
| 23 | Digital applications can help me to better prepare for a consultation with my doctor. | 0.676 | 9 (2.6%) |
| Excluded Variables due to low factor loadings < 0.4 | | | |
| 4 | I would be willing to pay for digital applications. | -0.305 | 4 (1.2%) |
| 24 | Digital applications can help to improve the doctor-patient communication. | 0.337 | 10 (2.9%) |
| 25 | Digital applications reduce the personal contact to my physician. | -0.204 | 10 (2.9%) |
| 12 | My attending physician would recommend digital applications to me. | 0.252 | 12 (3.5%) |
| 8 | I would trust my doctor's recommendations regarding digital applications. | 0.377 | 7 (2.0%) |
| 7 | I find it difficult to distinguish between serious and dubious digital applications. | -0.383 | 10 (2.9%) |
| Kaiser-Meyer-Olin Measure of Sampling Adequacy: 0.90  Bartlett’s Test of Sphericity: X² (df: 171) = 3335, p < 0.001  Method of rotation: Oblimin | | | |

*Polarity was retained for this factor; therefore, positive loadings still indicate higher digital competence (e.g., the item “I have excellent skills in dealing with digital media” loads positively, so greater agreement reflects greater digital competence).

Figure S1 Screeplot of the Exploratory Factor Analysis

Table S5 Total Variance Explained and Eigenvalues of Identified Factors

| Factor | Eigenvalue | % of variance explained | Accumulated % of variance explained |
| --- | --- | --- | --- |
| 1 | 7.409 | 38.995 | 38.995 |
| 2 | 2.196 | 11.556 | 50.551 |
| 3 | 1.273 | 6.699 | 57.250 |
| 4 | 1.142 | 6.011 | 63.261 |
| 5 | 0.981 | 5.161 | 68.423 |
| 6 | 0.935 | 4.919 | 73.342 |
| 7 | 0.676 | 3.560 | 76.902 |
| 8 | 0.633 | 3.331 | 80.233 |
| 9 | 0.545 | 2.867 | 83.100 |
| 10 | 0.504 | 2.655 | 85.755 |
| 11 | 0.463 | 2.436 | 88.192 |
| 12 | 0.428 | 2.255 | 90.447 |
| 13 | 0.365 | 1.923 | 92.369 |
| 14 | 0.314 | 1.653 | 94.022 |
| 15 | 0.291 | 1.529 | 95.551 |
| 16 | 0.289 | 1.521 | 97.073 |
| 17 | 0.204 | 1.071 | 98.144 |
| 18 | 0.181 | 0.954 | 99.098 |
| 19 | 0.171 | 0.902 | 100.000 |
| 20 | 0.265 | 1.062 | 96.316 |
| 21 | 0.244 | 0.977 | 97.293 |
| 22 | 0.207 | 0.827 | 98.120 |
| 23 | 0.178 | 0.710 | 98.830 |
| 24 | 0.166 | 0.664 | 99.494 |
| 25 | 0.126 | 0.506 | 100.000 |
