# Supplementary material for: Attitudes of dermatological patients towards digital health interventions: a cross‐sectional survey and cluster analysis
Source: J Dtsch Dermatol Ges. 2026 Apr 16;24(9):e1264–74. doi: 10.1111/ddg.70010 (PMC13551282; doi:10.1111/ddg.70010)
Supplement: Supplementary file 2 [file DDG-24-e1264-s002.docx]

**Online Supplement S4**

Table S6: Sociodemographics of study sampling by indication in comparison to (dermatological) population

|  | **Within our study** | | | **Prevalence in German population** | | |
| --- | --- | --- | --- | --- | --- | --- |
| **Indication**^1^ | % | % female | Mean age | % | % female | Mean age |
| **Psoriasis**^1^ | 39.0 | 59.2 | 60.2 | 2.5 | 43.4 | 43.2 |
| **Atopic dermatitis** | 23.3 | 88.6 | 47.6 | 3.5 | 43.2 | 43.2 |
| **Vitiligo**^3^ | 9.0 | 58.1 | 52.0 | 0.52 | 43.5 | 43.1 |
| **Skin cancer**^2^ | 7.6 | 88.5 | 55.0 | MM*: 0.22  NMSC**: 3.1 | 41.5 | 43.0 |
| **Hidradenitis**^1^ **suppurativa** | 21.2 | 86.3 | 43.8 | 1.0-2.0 | 47.7 | 43.6 |

*MM: Malignes Melanom

**NMSC: Nicht-melanozytärer Hautkrebs

^1^Hansen I, Augustin M, Schäfer I, Mohr N: Epidemiologie von Hautkrankheiten in Deutschland: systematische Literaturanalyse des aktuellen Forschungsstands – Teil 2: nichtinfektiöse Hauterkrankungen [Epidemiology of Skin Diseases in Germany: Systematic Review of the Current State of Research - Part 2: Non-infectious Skin Diseases]. J Dtsch Dermatol Ges 2022; 20 (4): 445-457.

^2^Hansen I, Augustin M, Schäfer I, Mohr N: Epidemiologie von Hautkrankheiten in Deutschland: systematische Literaturanalyse des aktuellen Forschungsstands - Teil 1: Tumorerkrankungen der Haut [Epidemiology of Skin Diseases in Germany: Systematic Review of the Current State of Research - Part 1: Cutaneous Tumor Diseases]. J Dtsch Dermatol Ges 2022; 20 (3): 257-271.

^3^Mohr N, Petersen J, Kirsten N, Augustin M: Epidemiology of Vitiligo – A Dual Population-Based Approach. Clinical Epidemiology 2021:13; 373-382.
