# Supplementary material for: Attitudes of dermatological patients towards digital health interventions: a cross‐sectional survey and cluster analysis
Source: J Dtsch Dermatol Ges. 2026 Apr 16;24(9):e1264–74. doi: 10.1111/ddg.70010 (PMC13551282; doi:10.1111/ddg.70010)
Supplement: Supplementary file 3 [file DDG-24-e1264-s003.docx]

# **Online Supplement S2 - Overview of the Items of the Survey**

## 1. **Acceptability of Digital Health Interventions**

**Explanation (German original):** Digitale Anwendungen bezeichnen im Folgenden jegliche digitalen Technologien, die zur Erreichung von gesundheitsbezogenen Zielen eingesetzt werden können.

**Explanation (English translation):** In the following, digital applications refer to any digital technologies that can be used to achieve health-related goals.

Each Item (see Table 1) was rated on a 5-Point Likert scale:

**German original:** (5) Stimme völlig zu; (4) Stimme eher zu; (3) Unentschieden; (2) Stimme eher nicht zu; (1) Stimme gar nicht zu

**English translation:** (5) Strongly agree; (4) Agree; (3) Neither; (2) Disagree; (1) Strongly disagree

Table S1 - Developed Items Related to the Acceptability of Digital Health Interventions

| **Nr** | **English translation** | **German original** |
| --- | --- | --- |
| Quest. | To what extent do you agree with the following statements? | Inwieweit stimmen Sie den folgenden Aussagen zu? |
| 1 | I could imagine using more digital applications for my condition. | Ich könnte mir vorstellen vermehrt digitale Anwendungen für meine Erkrankung zu nutzen. |
| 2 | I would use digital applications regularly if they were recommended to me. | Ich würde digitale Anwendungen regelmäßig nutzen, wenn Sie mir empfohlen werden würden. |
| 3 | I would recommend digital applications to other patients with a similar condition. | Ich würde digitale Anwendungen anderen Patienten mit einer ähnlichen Erkrankung empfehlen. |
| 4 | I would be willing to pay for digital applications. | Ich wäre bereit für digitale Anwendungen zu zahlen. |
| 5 | It would be very easy for me to use a digital application. | Mir würde es sehr leichtfallen eine digitale Anwendung zu nutzen. |
| 6 | I could easily integrate digital applications in my daily routines. | Ich könnte digitale Anwendungen einfach in meinen Alltag integrieren. |
| 7 | I find it difficult to distinguish between serious and dubious digital applications. | Ich finde es schwierig seriöse von unseriösen digitalen Anwendungen zu unterscheiden. |
| 8 | I would trust my doctor's recommendations regarding digital applications | Ich würde den Empfehlungen meines behandelnden Arztes in Bezug auf digitale Anwendungen vertrauen. |
| 9 | I would trust the recommendations of other patients regarding digital applications. | Ich würde den Empfehlungen von anderen Patienten in Bezug auf digitale Anwendungen vertrauen. |
| 10 | I would trust the recommendations of a patient self-help association with regard to digital applications. | Ich würde den Empfehlungen eines Patientenselbsthilfeverbandes in Bezug auf digitale Anwendungen vertrauen. |
| 11 | People in my immediate environment would be in favor of me using digital applications. | Personen in meinem direkten Umfeld würden es befürworten, wenn ich digitale Anwendungen nutzen würde. |
| 12 | My attending physician would recommend digital applications to me | Mein behandelnder Arzt würde den Einsatz digitaler Anwendungen befürworten. |
| 13 | I could imagine discussing digitally collected data with the attending physician | Ich könnte mir vorstellen von mir digital gesammelte Daten mit meinem behandelnden Arzt zu besprechen. |
| 14 | I should be able to release my collected digital data individually to my attending physician. | Meine gesammelten digitalen Daten müssten von mir individuell für meinen behandelnden Arzt freigegeben werden können. |
| 15 | I am worried that collected data will be misused. | Ich mache mir Sorgen, dass erhobene Daten missbraucht werden. |
| 16 | Digital applications can make me feel insecure about my illness. | Digitale Anwendungen können mich in Hinblick auf meine Erkrankung verunsichern. |
| 17 | Digital applications can make the disease too much the focus of my everyday life. | Digitale Anwendungen können die Erkrankung zu sehr zum Mittelpunkt meines Alltags machen. |
| 18 | Digital applications can save unnecessary journeys for me as a patient. | Digitale Anwendungen können unnötige Wege für mich als Patient einsparen. |
| 19 | Digital applications can be a useful addition to my existing care | Digitale Anwendungen können eine sinnvolle Ergänzung zu meiner bestehenden Versorgung sein. |
| 20 | Digital applications can strengthen confidence in my therapy | Digitale Anwendungen können das Vertrauen in meine Therapie stärken. |
| 21 | Digital applications can help me to deal more with my illness | Digitale Anwendungen können mir helfen mich mehr mit meiner Erkrankung auseinanderzusetzen. |
| 22 | Digital applications can help me to strengthen my personal responsibility in treatment | Digitale Anwendungen können mir helfen meine Eigenverantwortung in der Behandlung zu stärken. |
| 23 | Digital applications can help me to better prepare for a consultation with my doctor | Digitale Anwendungen können mir helfen mich besser auf eine Sprechstunde mit meinem Arzt vorzubereiten. |
| 24 | Digital applications can help to improve the doctor-patient communication | Digitale Anwendungen vermindern den persönlichen Kontakt zu meinem behandelnden Arzt. |
| 25 | Digital applications reduce the personal contact to my physician | Digitale Anwendungen können helfen die Arzt-Patienten-Kommunikation zu verbessern. |
| 26 | I have excellent skills in dealing with digital media (e.g., PC, smartphone, tablet) | Ich habe exzellente Fähigkeiten im Umgang mit digitalen Medien (z.B. PC, Smartphone, Tablet). |
| 27 | I feel safe when using digital/online applications | Ich fühle mich sicher bei der Nutzung digitaler/Online-Anwendungen. |
| 28 | The Internet is something threatening for me | Das Internet ist etwas Bedrohliches für mich. |
| 29 | I am afraid of making an uncorrectable mistake when using digital/online applications | Ich habe Angst, bei der Nutzung digitaler/Online-Anwendungen einen nicht korrigierbaren Fehler zu machen. |
| 30 | I know where I can find useful health information on the Internet. | Ich weiß, wo ich im Internet nützliche Gesundheitsinformationen finden kann. |
| 31 | I can distinguish reliable from questionable information on the Internet | Ich kann im Internet zuverlässige von fragwürdigen Informationen unterscheiden. |

## 2. **Current Use of Available DHIs**

All items were answered with “Yes” / “No” or “I do not know”.

Table S2 Current Use of Available DHIs

| **Nr** | **English translation** | **German original** |
| --- | --- | --- |
| Quest. | Which of the following digital applications have you used for your skin condition in the last 12 months? | Welche der folgenden digitalen Anwendungen haben Sie für Ihre Hauterkrankung in den letzten 12 Monaten genutzt? |
| 1 | Live-interactive video consultation with your treating doctor | Live Online-Videosprechstunde mit Ihrem behandelnden Arzt |
| 2 | Online dermatologists offer or practice (written consultation after digital transmission of your skin problem, e.g., with the help of photos) (S&F Teledermatology) | Online-Hautarztangebot bzw.-praxis (Schriftliche Beratung nach digitaler Übermittlung Ihres Hautproblems, z.B. mit Hilfe von Fotos) |
| 3 | E-mail or e-mail-like application for communicating with your doctor | E-Mail oder E-Mail-ähnliche Anwendung zur Kommunikation mit Ihrem Arzt |
| 4 | Instant messenger (e.g., WhatsApp or similar) to communicate with your doctor | Instant Messenger (z.B. WhatsApp oder ähnlich) zur Kommunikation mit Ihrem Arzt |
| 5 | Sharing experiences with patients (e.g., via email, Facebook, Instagram, forums) | Erfahrungsaustausch mit Patienten (z.B. via E-Mail, Facebook, Instagram, Foren) |
| 6 | Application for medication reminders | Anwendung zur Erinnerung an die Medikation |
| 7 | Application to remind you of appointments | Anwendung zur Erinnerung an Termine |
| 8 | Official electronic patient record of your health insurance company | Elektronische Patientenakte Ihrer Krankenkasse |
| 9 | Application for documenting the course of the disease (regular entries in a digital patient diary) | Anwendung zur Dokumentation des Krankheitsverlaufes (regelmäßige Eintragungen in digitales Patiententagebuch) |
| 10 | Online information and education portals | Online-Informations- und Aufklärungsportale |
| 11 | Online pollen forecast | Online-Pollenflugvorhersage |

## 3. **Potential Future Use of DHIs**

Each Item (see Table 3) was rated on a 5-Point Likert scale:

**German original:** (5) Stimme völlig zu; (4) Stimme eher zu; (3) Unentschieden; (2) Stimme eher nicht zu; (1) Stimme gar nicht zu

**English translation:** (5) Strongly agree; (4) Agree; (3) Neither; (2) Disagree; (1) Strongly disagree

Table S3 Potential Future Use of DHIs

| **Nr** | **English translation** | **German original** |
| --- | --- | --- |
| Quest. | To what extent do you agree with the following statements? | Ich könnte mir vorstellen … |
| 1 | … eine Live Online-Videosprechstunde mit meinem behandelnden Arzt zu nutzen. | ... to use a live-interactive video consultation with my attending physician. |
| 2 | … eine Online-Hautarztpraxis bzw. -angebot (Schriftliche Beratung nach digitaler Übermittlung Ihres Hautproblems) zu nutzen. | ... to use an online dermatology practice or offer (written consultation after digital transmission of your skin problem). (S&F Teledermatology) |
| 3 | … per E-Mail oder mit einer E-Mail-ähnlichen Anwendung mit meinem behandelnden Arzt zu kommunizieren. | ... to communicate with my treating doctor by e-mail or with an e-mail-like application. |
| 4 | … eine Anwendung (z.B. WhatsApp oder ähnlich) zur Kommunikation mit meinem behandelnden Arzt zu nutzen. | ... to use an application (e.g. WhatsApp or similar) to communicate with my treating doctor. |
| 5 | … Erfahrungen mit Patienten digital auszutauschen (z.B. via E-Mail, Facebook, Instagram, Foren). | ... to share experiences with patients digitally (e.g. via E-Mail, Facebook, Instagram, forums). |
| 6 | … eine Anwendung zur Erinnerung an die Medikation zu nutzen. | ... to use an application for medication reminders. |
| 7 | … eine Anwendung zur Erinnerung an Termine zu nutzen. | ... use an application to remind me of appointments. |
| 8 | … die elektronische Patientenakte meiner Krankenkasse zu nutzen. | ... to use the electronic patient file of my health insurance company. |
| 9 | … eine Anwendung zur Dokumentation des Krankheitsverlaufes (regelmäßige Eintragungen in digitales Patiententagebuch) zu nutzen. | ... use an application to document the course of my illness (regular entries in a digital patient diary). |
| 10 | … Online Informations- und Aufklärungsportale zu nutzen. | ... to use online information and education portals. |
| 11 | … Online-Pollenflugvorhersagen zu nutzen. | ... to use online pollen forecasts. |
