# Supplementary material for: Attitudes of dermatological patients towards digital health interventions: a cross‐sectional survey and cluster analysis
Source: J Dtsch Dermatol Ges. 2026 Apr 16;24(9):e1264–74. doi: 10.1111/ddg.70010 (PMC13551282; doi:10.1111/ddg.70010)
Supplement: Supplementary file 4 [file DDG-24-e1264-s004.docx]

# **Supplement 5 – Additional Results**

Table S7 Item-By-Item Agreement Rates Overall Group (n = 344)

| Item | Text | Missing | Agree / Fully Agree  (n = 344) |
| --- | --- | --- | --- |
| Factor 1: Acceptability of DHIs and Trust” | | | |
| 1 | I could imagine using more digital applications for my condition. | 6 (1.7%) | 260 (76.9%) |
| 3 | I would recommend digital applications to other patients with a similar condition. | 7 (2.0%) | 221 (65.6%) |
| 2 | I would use digital applications regularly if they were recommended to me. | 4 (1.2%) | 260 (76.5%) |
| 13 | I could imagine discussing digitally collected data with the attending physician | 3 (0.9%) | 252 (75.2%) |
| 14 | I should be able to release my collected digital data individually to my attending physician. | 6 (1.7%) | 266 (79.2%) |
| 18 | Digital applications can save unnecessary journeys for me as a patient. | 7 (2.0%) | 264 (78.3%) |
| 11 | People in my immediate environment would be in favor of me using digital applications. | 6 (1.7%) | 108 (32.0%) |
| 19 | Digital applications can be a useful addition to my existing care. | 6 (1.7%) | 279 (82.5%) |
| 10 | I would trust the recommendations of a patient self-help association with regard to digital applications. | 6 (1.7%) | 273 (79.4%) |
| 9 | I would trust the recommendations of other patients regarding digital applications. | 5 (1.5%) | 158 (46.6%) |
| Factor 2: Patients’ Digital Health Competencies | | | |
| 26 | I have excellent skills in dealing with digital media (e.g., PC, smartphone, tablet). | 8 (2.3%) | 239 (71.1%) |
| 27 | I feel safe when using digital/online applications. | 7 (2.0%) | 277 (82.2%) |
| 5 | It would be very easy for me to use a digital application. | 5 (1.5%) | 246 (72.6%) |
| 31 | I can distinguish reliable from questionable information on the Internet. | 6 (1.7%) | 251 (74.3%) |
| 6 | I could easily integrate digital applications in my daily routines. | 6 (1.7%) | 251 (74.3%) |
| 30 | I know where I can find useful health information on the Internet. | 5 (1.5%) | 294 (86.7%) |
| Factor 3: Digital Health Insecurities | | | |
| 16 | Digital applications can make me feel insecure about my illness. | 8 (2.3%) | 148 (43.9%) |
| 17 | Digital applications can make the disease too much the focus of my everyday life. | 4 (1.2%) | 81 (24.2%) |
| 15 | I am worried that collected data will be misused. | 6 (1.7%) | 148 (43.9%) |
| 28 | The Internet is something threatening for me. | 11 (3.2%) | 16 (4.8%) |
| 29 | I am afraid of making an uncorrectable mistake when using digital/online applications. | 9 (2.6%) | 30 (9.0%) |
| Factor 4: Positive Impact of DHIs | | | |
| 21 | Digital applications can help me to deal more with my illness | 8 (2.3%) | 176 (52.4%) |
| 22 | Digital applications can help me to strengthen my personal responsibility in treatment. | 9 (2.6%) | 190 (56.7%) |
| 20 | Digital applications can strengthen confidence in my therapy. | 9 (2.6%) | 175 (52.4%) |
| 23 | Digital applications can help me to better prepare for a consultation with my doctor. | 9 (2.6%) | 232 (69.3%) |
| Excluded Variables due to low factor loadings < 0.4 | | | |
| 4 | I would be willing to pay for digital applications. | 4 (1.2%) | 64 (18.8%) |
| 24 | Digital applications can help to improve the doctor-patient communication. | 10 (2.9%) | 180 (53.9%) |
| 25 | Digital applications reduce the personal contact to my physician. | 10 (2.9%) | 164 (49.1%) |
| 12 | My attending physician would recommend digital applications to me. | 12 (3.5%) | 55 (16.6%) |
| 8 | I would trust my doctor's recommendations regarding digital applications. | 7 (2.0%) | 268 (79.5%) |
| 7 | I find it difficult to distinguish between serious and dubious digital applications. | 10 (2.9%) | 103 (30.4%) |

Table S8 Usage of DHI in the Last 12 Months (n = 344)

|  | Missing | Used in the last 12 Months (N; N%) |
| --- | --- | --- |
| Live-interactive teledermatology | 4 (1.2%) | 7 (2.1%) |
| Instant messaging with attending physician | 5 (1.5%) | 9 (2.6%) |
| Store-and-forward teledermatology | 4 (1.2%) | 14 (4.1%) |
| Electronic patient diary | 5 (1.5%) | 18 (5.2%) |
| Official electronic patient record | 7 (2.0%) | 45 (13.1%) |
| Electronic medication reminder | 8 (2.3%) | 66 (19.2%) |
| Online pollen count forecast | 7 (2.0%) | 108 (31.4%) |
| Use of online application for exchange among patients | 4 (1.2%) | 138 (40.1%) |
| E-Mail communication with attending physician | 4 (1.2%) | 98 (28.5%) |
| Appointment reminder | 5 (1.5%) | 184 (53.5%) |
| Online information and education portals | 7 (2.0%) | 219 (63.7%) |

Table S9 Potential Future Usage of DHIs (n = 344)

| Type of intervention | Missing | Agree / Fully Agree (N; N%) |
| --- | --- | --- |
| Patients that can imagine to use a DHI in the future: | | |
| Instant messaging with attending physician | 7 (2.0%) | 180 (52.3%) |
| Online pollen count forecast | 11 (3.2%) | 189 (54.9%) |
| Use of online application for exchange among patients | 5 (1.5%) | 200 (58.1%) |
| Store-and-forward teledermatology | 5 (1.5%) | 213 (61.9%) |
| Electronic medication reminder | 9 (2.6%) | 217 (63.1%) |
| Electronic patient diary | 9 (2.6%) | 234 (68.0%) |
| Live-interactive teledermatology | 5 (1.5%) | 235 (68.3%) |
| Official electronic patient record | 10 (2.9%) | 237 (68.9%) |
| E-Mail communication with attending physician | 6 (1.7%) | 252 (73.3%) |
| Appointment reminder | 9 (2.6%) | 276 (80.2%) |
| Online information and education portals | 9 (2.6%) | 294 (85.5%) |
